# Molecular and cultural evidence of corrosive microorganisms in an offshore oil field in the Arctic (Russia)

**DOI:** 10.1128/msystems.01161-25

**Published:** 2025-10-24

**Authors:** Diyana S. Sokolova, Anna A. Kruglova, Ekaterina M. Semenova, Tatiana A. Mayorova, Andrey V. Mardanov, Tamara N. Nazina

**Affiliations:** 1 Winogradsky Institute of Microbiology, Research Center of Biotechnology, Russian Academy of Sciences, Moscow, Russia; 2 Independent Researcher, Saint Petersburg, Russia; 3 Institute of Bioengineering, Research Center of Biotechnology, Russian Academy of Sciences, Moscow, Russia; Third Institute of Oceanography Ministry of Natural Resources, Xiamen, China

**Keywords:** offshore oil field, microbial communities, high-throughput sequencing, quantitative polymerase chain reaction (qPCR), thermophiles, sulfate-reducing bacteria (SRB), methanogens

## Abstract

The use of seawater with a high sulfate content for water-flooding of oil reservoirs contributes to the growth of sulfate-reducing bacteria (SRB), producing sulfide, which causes oil souring, corrosion of steel equipment, and environmental problems during oil production and refining. The purpose of this work was to determine the composition of microorganisms and the physicochemical parameters of the production and injection water of the Prirazlomnoye offshore high-temperature oil field in order to improve our understanding of the corrosion-active microorganisms. High-throughput sequencing of V3–V4 fragments of the 16S rRNA gene, quantitative PCR of bacteria and archaea, and cultural and analytical methods were used in the work. SRB, fermentative and syntrophic bacteria, and methanogenic archaea capable of participating in the processes of general and pitting corrosion of steel equipment were found in water samples. The production water contains sulfate, sulfide, and thermophilic SRB (*Thermacetogenium* and *Desulfonauticus*). Mixing of the produced water with seawater leads to its cooling and emergence of mesophilic SRB (*Desulfobacter* and *Desulfogranum*) in the water treatment system. Residual oil hydrocarbons and sulfides can be oxidized in the water treatment system, and the resulting metabolites serve as electron donors and acceptors for fermentative sulfidogenic bacteria (genera *Caminicella*, *Kosmotoga*, *Petrotoga*, and *Geotoga*). Sulfate-reducing and methanogenic enrichments from reservoir water produce sulfide and methane, respectively, receiving electrons from Fe^0^ in the absence of other sources of H_2_, which can contribute to pitting corrosion. This study allows for improving the ways to control sulfidogens and expands understanding of the microbial diversity of oil reservoirs.

**IMPORTANCE:**

Oil production from oil reservoirs with sulfate-containing formation water and injection seawater is accompanied by the appearance of sulfide in oil production, which increases the cost of oil refining and enhances corrosion processes of steel equipment. In this work, the physicochemical conditions and the composition of microorganisms in the produced and injected seawater at the Prirazlomnoye oil field (Russia) are investigated. The biocides used at the oil field are ineffective in suppressing sulfate-reducing bacteria (SRB), which are considered the main agents of microbial corrosion. It has been shown that not only SRBs but also fermenting bacteria inhabiting the oilfield were capable of producing sulfide. Enrichment cultures of autotrophic SRBs and methanogens capable of receiving electrons directly from Fe^0^ with the production of sulfide and methane, respectively, were obtained. The new scientific information obtained on microbial communities of oil reservoirs will make it possible to improve methods for monitoring corrosive microorganisms and selecting biocides.

## INTRODUCTION

Corrosion damage to metal structures and aggregates used for the extraction, processing, and transportation of oil and gas is determined by the influence of technological, physicochemical, and microbiological factors (1–3). The degree of aggressive impact of field environments on oil and gas equipment made of carbon steel depends on the presence and concentration of aggressive components in them, the number of suspended particles, the velocity of reservoir fluids, temperature, mineralization, and pH of the aqueous phase, and the presence of corrosive microorganisms (3, 4). Ignoring the biological contamination of pumped liquids can lead to the development of biocorrosion of equipment, accompanied by local pitting and generalized corrosion of equipment, deterioration of the filtration properties of the reservoir, and a decrease in the effectiveness of the reagents used in the oil production process (5–7).

The corrosion of steel equipment used for oil recovery was the first problem that stimulated the development of petroleum microbiology. In 1926, Bastin and co-authors (8) in the USA and Ginzburg-Karagicheva (9) in the former Soviet Union simultaneously discovered the accumulation of hydrogen sulfide in sulfate-containing reservoir waters and made an assumption about the microbial nature of this process. Currently, there is no doubt about the leading role of sulfate-reducing bacteria in the corrosion of oilfield equipment. However, information about the microorganisms that cause corrosion has significantly expanded (10–14).

In addition to sulfate-reducing bacteria (SRB), which grow over a wide temperature range, corrosive prokaryotes also include thermophilic sulfate-reducing archaea (SRA) (15). It has been shown that sulfide can also be produced by a wide range of fermentative bacteria and archaea, which reduce oxidized sulfur compounds other than sulfate (sulfite, thiosulfate, and elemental sulfur) to sulfide (16–19). Acid-producing bacteria that ferment organic substrates in the absence of an external electron acceptor (12, 20), metal-reducing bacteria of the genera *Shewanella* and *Geobacter* (21), bacteria that oxidize sulfur and iron (14), acetogenic bacteria (22), and methanogenic archaea (23, 24) also have corrosive activity. Many anaerobic microorganisms that consume hydrogen (acetogens, sulfate-reducers, and methanogens) contribute to corrosion in the absence of organic substrates by direct transfer of electrons from iron to the microbial cell (22, 25–28).

The accumulation of hydrogen sulfide/sulfide in reservoir waters leads to “souring” of oil (1). Large economic losses are observed in deposits with moderate temperature and salinity of reservoir water, where conditions are favorable for the development of mesophilic sulfidogens (29), whereas in reservoirs with temperatures around 65°C–70°C, sulfate reduction proceeds at a slower rate and is not recorded at 80°C (30). Corrosion of steel equipment is particularly accelerated on oil platforms, where seawater with high sulfate content is injected to maintain the reservoir pressure (7, 31–35).

Injection of various biocides, as well as nitrate, nitrite, and (per)chlorate flooding technologies is used to suppress the growth of SRB (36, 37). Despite the selective action of some biocides, not all sulfidogenic bacteria are sensitive to them (38–40). Bacteria exist in the natural environment in the form of complexly organized communities—biofilms enclosed in an exopolymer matrix containing channels through which electron donors and acceptors flow and metabolic products are excreted (41–43). SRB biofilms mainly consist of proteins with a low content of exopolysaccharides (44). The formation of multi-species microbial biofilms further increases their resistance to growth inhibitors. A comparison of the survival of sulfidogenic bacteria in the presence of different biocides demonstrated greater stability of biofilms relative to planktonic cells (35). To select the ways to suppress corrosion of steel equipment, information on the composition and functional activity of microorganisms in production and injection water is required.

In this work, we analyzed microbial diversity in water samples collected at the Prirazlomnoye offshore oil field, located in the Arctic region (Russia). Since the beginning of development in 2013, the oil field has been operated using flooding with a mixture of seawater and reservoir water separated from oil. Oil production is carried out from an offshore platform in compliance with high environmental standards. All extracted oil, reservoir water, and domestic wastewater are treated on site in the water and oil treatment system (45). Sulfide/H_2_S is present in the produced and injection water and in the gas, which causes corrosion of steel equipment. Biocides based on formaldehyde mixed with methanol are used in the field to protect against SRB, but this does not lead to a significant decrease in the content of sulfide and the number of SRB in the water treatment system.

The purpose of this work was to determine the physicochemical parameters and microbial diversity in water from different sampling sites of the production/injection water treatment system, as well as to identify possible producers of hydrogen sulfide and other potential agents of microbial corrosion at the Prirazlomnoye oil field.

## MATERIALS AND METHODS

### Research subjects and sampling

The water samples were collected in June 2023 and January and November 2024 at the Prirazlomnoye offshore oil field (Nenets Autonomous region, Russia), which is the only field operating in Russia for the production of hydrocarbons on the Arctic shelf. Oil production has been underway since December 2013 from an offshore ice-resistant oil-producing stationary platform, which was built specifically for the Prirazlomnoye field. The platform is located in the Pechora Sea. All technological operations are performed on the platform, including drilling wells, extraction, storage, and shipment of oil to tankers, and a residential module is also located on the platform. The coproduced water is pumped back into the oil reservoirs after appropriate treatment with various biocides. More polluted wastewater is collected in separate containers, pumped onto a special vessel, and transported ashore for disposal.

Productive horizons lie at a depth of 2,300–2,700 m (45). The strata are represented by relatively dense carboniferous limestones with an average porosity of 15.6%–21.7% and a permeability of 0.05–0.4 µm^2^. The temperature of oil reservoirs is 68°C, whereas in the water treatment system, it decreases to 28°C–32°C. Water pumped into the reservoir has a temperature of 30°C–40°C. The extracted oil belongs to a new type called Arctic Oil (ARCO). The density of crude oil is about 0.910–0.970 g·cm^−3^. Oil is classified as heavy, bituminous, and highly viscous, with high sulfur content and low paraffin content. The composition of the oil is dominated by resins over paraffins and asphaltenes, with mechanical impurities in the range of 0.0007%–0.2880%. Oil is unstable in terms of asphaltenes and low-paraffin. The gas factor varies from 25 to 47 m^3^·m^−3^. Coproduced gas contains up to 0.4% (vol/vol) H_2_S, and the sulfur content in oil is 2.3% (wt/vol). Reservoir water is of the chloride-calcium type, has a total salinity of up to 35–45 g L^−1^, and high sulfate content.

The produced water, separated from the oil, is fully used for back injection into the reservoir to maintain the reservoir pressure. Water samples were taken from different tanks of the oil and reservoir water extraction and treatment system and delivered to the laboratory within 20 min for enumeration of the SRB on nutrient media. The samples for chemical and molecular analysis were collected separately in sterile vials, hermetically sealed without air bubbles. The samples for molecular studies were collected in sterile glass vials with a volume of 1 L and fixed with ethanol (70:30, vol/vol) at the time of sampling. In the laboratory, water samples were filtered through membrane filters with a pore size of 0.22 µm (Millipore, USA). Before the analysis, the filters were dried at room temperature and stored at –20°C. The list of water samples examined is shown in Table 1, and the sampling sites are shown in Fig. S1.

**TABLE 1:** List of the production and injection water samples and liquids from the water treatment system

No.	Sample	Description	pH	Temperature, °C	Dissolved gas content, mg L^−1^		
					O_2_	CO_2_	H_2_S
1	V20002	Production water collected after the separator of oil-water emulsion V20002	7.3	49	0–0.05	40	51.1
2	V48002a	Production water collected before the equalization tank V48002	7.2	53	0–0.05	30–35	48.4
3	V48002b	Production water collected after the equalization tank V48002	7.0	50	0–0.05	40	0.7
4	Z43003	Water collected before the gas flotation device Z43003	7.3	22	1	18–20	4.8
5	Z49002	Water collected after fine filters Z49002	7.3	56	0.1	16–18	9.9
6	V49001	Injection water collected after the vacuum deaerator V49001	7.6	ND^*b*^	0–0.05	10–11	11.9
7	T53001	Wastewater collected after the tank of safe drains Т53001	6.8	25–50	0.7–0.8	20–25	25.2
8	T53004	Wastewater collected after the tank of dangerous drains Т53004	7.1	25–50	1	25	7.5
9	T43006	Wastewater collected after the filter backwash tank Т43006	7.9	50	–^*a*^	–^*a*^	–^*a*^
10	FCO	Water collected after the coarse filter FCO	7.7	38	2–3	<10	42.2
11	P22004	Water collected after pump Р22004	7.1	38	1-2	<10	1.0

^*a*^ Could not be determined because the liquid was dark in color.

^*b*^ “ND” indicates no data.

### DNA isolation and sequencing of the V3–V4 fragments of the 16S rRNA genes

Microbial biomass collected on the membrane filters was washed off with a lysing solution (0.15 M NaCl and 0.1 M Na_2_EDTA, pH 8.0) and used for DNA extraction with the DNeasy PowerSoil Pro kit (Qiagen, Germany), according to the manufacturer’s recommendations. Microbial composition was determined by construction of libraries of the V3–V4 regions of the 16S rRNA genes as described previously (35). The template of DNA isolated from each specimen was used for PCR amplification with a pair of primers: 341F (5'-CCTAYGGGDBGCWSCAG-3') and 806R (5'-GGACTACNVGGGTHTCTAAT-3') (46). High-throughput sequencing of amplified V3–V4 fragments of the 16S rRNA genes was performed using the MiSeq system (Illumina, San Diego, CA, USA) with a MiSeq Reagent Kit v3 (600 cycles) (Illumina, United States) as recommended by the manufacturer. Libraries of 16S rRNA gene fragments from samples collected in June 2023, designated _1, in January 2024, designated _2, and in November 2024, designated _3.

### Data analysis

The 16S rRNA gene fragments in all libraries were clustered together into OTUs (operational taxonomic units) at 97% identity using USEARCH (47). The OTUs were taxonomically identified through searches against the SILVA v.138 rRNA sequence database using the VSEARCH v. 2.14.1 algorithm (48). Chao1 and Shannon diversity indices were calculated using USEARCH v.11 (47), and sequences not assigned to a particular phylum were excluded from further analysis. The ClustVis online resource was used to create a principal component analysis (PCA) graph and heat maps of community members at the genus level (49). The functional characteristics of the studied bacterial communities were determined based on the taxonomic composition (at the genus level) when compared with the genomic data from the KEGG database (50) using the Global Mapper module of the iVikodak program (51). Maps of metabolic pathways were obtained using the KEGG Database (52). Canonical correlation analysis (CCA) was performed in the PAST 4.03 program (53).

### Quantification of bacterial and archaeal 16S rRNA genes in the water samples

The number of 16S rRNA genes of *Bacteria* and *Archaea* was determined by quantitative PCR (qPCR) method in real time using a DNA template and the following appropriate primer systems: Eub338F/Eub518R and Arch 967F/Arch-1060R for the determination of the bacterial and archaeal genes, respectively (54, 55). Real-time PCR using SYBR Green I technology was performed in a PCR buffer-RV (Syntol, Russia) in the presence of a passive reference dye ROX to normalize the fluorescence signal of the dye used in the reaction. Detection for each sample was carried out in a 3-fold repeat. The negative control (reaction mixture without a DNA template) was ddH_2_O (Syntol, Russia). Amplification was performed on a LightCycler96 DNA amplifier “Roche” (Germany) in compliance with the temperature-time profile of the reaction (54, 55). To calculate the number of bacterial and archaeal sequences in the analyzed samples, the signal received in a studied unknown sample was compared with a standard curve. A series of successive dilutions of the standard sample was used to construct the standard curves. The target PCR fragment, previously purified using the “Wizard SV Gel and PCR Clean-Up System” kit (Promega, USA) and subsequently cloned into a pGEM-T vector (Promega, USA), was used as a standard sample.

### Quantification of culturable prokaryotes and analytics

The number of culturable sulfate-reducing bacteria was determined via MPN (Most Probable Number) by inoculating 10-fold dilutions of the water samples into the liquid Postgate B medium containing per liter distilled water: 0.5 g KH_2_PO_4_; 1.0 g NH_4_Cl; 1.0 g CaSO_4_; 2.0 g MgSO_4_·7H_2_O; 5.0 g NaCl; and 3.5 g sodium lactate (56). After sterilization, this medium was supplemented with 1 g L^−1^ yeast extract and 0.5 g L^−1^ FeSO_4_·7H_2_O, and 5% aqueous NaHCO_3_ solution was used to establish the pH of the medium to 7.0–7.2. Then, the medium was reduced by 0.2 g Na_2_S·9H_2_O. Argon was used as the gas phase. The MPN experiments were carried out in triplicate, incubated at 30°C for 14 days, and the absence of growth was recorded after 30 days of incubation. Growth was assessed by sulfide production in the media with terminal dilutions. Sulfide was analyzed by the calorimetric method with *p*-phenylenediamine (57).

Anaerobic enrichment cultures of fermentative, sulfate-reducing, and methanogenic prokaryotes were obtained by inoculation of the production water sample V48002a_2 into a mineral medium (58) supplemented with various substrates and electron acceptors. Mineral medium contained per liter distilled water 3.0 g MgCl_2_∙6H_2_O; 0.3 g KCl; 0.15 g CaCl_2_; 0.3 g NH_4_Cl; 0.2 g KH_2_PO_4_; 20.0 g NaCl; and 2.5 g NaHCO_3_ (58). The medium was supplemented with solutions of trace elements (59) and vitamins (60), 1 mL per 1 L each. Peptone, glucose, and pyruvate were added at a final concentration of 5.0 g L^−1^, lactate and acetate 3.5 g L^−1^, and oil 0.5% (vol/vol). Na_2_SO_4_ (4.0 g L^−1^), Na_2_S_2_O_3_·5H_2_O (5.0 g L^−1^), and elemental sulfur (10.0 g L^−1^) were used as electron acceptors. The medium for sulfate-reducing prokaryotes was amended with Na sulfate (4.0 g L^−1^) and reduced by adding 0.2 g L^−1^ Na_2_S·9H_2_O, and the medium for methanogens did not contain sulfates and was reduced with 0.5 g L^−1^ Na_2_S·9H_2_O. A steel needle was inserted into the methanogen and SRB media that did not contain electron donors. The pH value of the medium was 7.0–7.2. The experiments were carried out at two temperatures (48°C and 55°C) in triplicate each for 30 days in stationary conditions in the dark.

Methane, hydrogen, and CO_2_ in the headspace were determined by gas chromatography (35). Scanning electron microscopy of enrichment cultures was performed using a scanning electron microscope (Quattro S, Thermo Fisher Scientific, Brno-Chernivtsi, Czech Republic) at an accelerating voltage of 15 kV as described previously (61). The content of inorganic anions was determined using a Staier ion chromatograph (Aquilon, Russia) with a connected conductometric detector. Previously, the water sample was centrifuged to remove the cells. Separation was performed on a Dionex IonPac AS22 column (Thermo Scientific, USA) in an isocratic mode with a buffer eluent of 4.5 mM carbonate/1.4 mM sodium bicarbonate and a flow rate of 1.0 ml/min. Concentrations of sodium, potassium, calcium, and magnesium ions were analyzed using the Capel 104T capillary electrophoresis system (Lumex, St. Petersburg, Russia). Saint Petersburg, Russia) in accordance with the manufacturer’s protocols (62). The analyses were carried out in triplicate.

## RESULTS

### Physicochemical characteristics of the water samples and culturable SRB

The physicochemical and microbiological characteristics of samples from the water treatment system are shown in Table 2. Water samples had salinity in the range of 30.27–54.50 g L^−1^, pH range from 6.8 to 7.9, and were characterized by high sulfate content (from 1.87 to 3.43 g L^−1^). The water chemistry did not change significantly during the water flow along the production line from the separator V20002 to the injection water system V49001. Sulfide was present in the water samples. Its concentration was the highest (42.2–51.1 mg L^−1^) in the formation water from production well (V20002), in the water sampled after the coarse filter FCO, and in the sample collected before the production water equalization tank V48002 (Table 1). Despite the hypochlorite treatment of Z54004 domestic wastewater and its pumping into the T53001 reservoir, where it was treated with a biocide, the wastewater at the outlet of the T53001 reservoir contained 25.2 mg S^2–^ L^−1^.

**TABLE 2:** Physicochemical characteristics of the studies’ water samples and the number of culturable sulfate-reducing bacteria (June 2023)

Sample and sampling point	Total salinity, mg L^−1^	Content, mg L^−1^								SRB, cells·mL^−1^
		K^+^	Na^+^	Ca^2+^	Mg^2+^	Fe_total_	Cl^–^	НСО_3_^–^	SO_4_^2–^	
V20002	37,743.9	0	11,999.1	741.5	1,130.9	0	21,300	695.4	1,877.0	10^3^
V48002a	49,889.4	307.0	13,878.1	1,324.1	1,187.1	2.2	29,846.1	676.3	2,668.6	10
V48002b	54,504.8	332.4	15,516.6	1,272.1	1,400.2	2.3	32,295.8	668.3	3,017.3	10
Z43003	48,805.1	397.6	13,643.9	586.7	1,515.5	0.7	29,074.3	146.1	3,440.4	10^5^
Z49002	48,793.6	350.4	13,602.0	792.4	1,504.7	1.2	28,899.8	265.9	3,377.4	10^5^
V49001	32,193.6	ND^*a*^	10,143	621.2	1,033.6	0	18,176	280.6	1,939.2	10^6^
Т53001	30,274.1	234.1	8,583.0	682.0	1,003.1	10.6	16,924.1	373.9	2,463.4	10^6^
Т53004	48,062.6	345.9	13,784.9	687.8	1,519.6	2.1	28,767.7	659.2	2,295.5	10
Т43006	50,267.2	498.1	12,977.3	333.4	1,205.7	4.0	31,077.4	1259.9	2,911.5	10
FCO	51,089.1	345.8	13,014.3	535.0	1,616.6	5.6	31,983.6	152.5	3,435.8	0
Р22004	46,403.9	346.8	13,702	505.2	1,556.0	1.4	28,015.2	161.1	2,116.3	0

^*a*^ “ND” indicates no data.

Culturable SRBs were not detected in the lactate-sulfate medium inoculated with water collected after the coarse filter FCO and pump P22004. This water was represented by ballast seawater, which subsequently enters the injection/production water treatment system. Bacteria were grown for 30 days at 30°C in accordance with the approved regulations. In water samples V48002_a, V48002_b, Т53004, and Т43006, SRB numbers did not exceed dozens of cells per 1 mL as counted by MPN in the lactate-sulfate medium. In production water separated from oil at the first separator V20002, the number of SRB was 10^3^ cells·mL^−1^ and increased to 10^5^–10^6^ cells·mL^−1^ in water samples Z43003, Z49002, V49001, and Т53001. A sharp increase in the degree of contamination with bacteria at the entrance of Z43003 was associated with the flow of wastewater from the reservoir of safe drains T53001 and from the P2204 pump that delivered seawater and ballast water from oil storage tanks. Reservoirs T53004 and T43006 receive various effluents for storage from 2 to 4 days, then wastewater from T53004 is pumped onto a ship and transported for further processing on shore, and wastewater from T43006 is removed into a slurry well.

### Microbial diversity

Using high-throughput V3–V4 sequencing of 16S rRNA gene fragments, the composition of microorganisms was determined in 28 water samples, taken from the injection and production water treatment system at different times at the Prirazlomnoye oil field. For each sample, 11,437–66,277 V3–V4 fragments were sequenced. As a result of clustering fragments with a 97% similarity level, from 2,204 to 30,469 OTUs per library were formed (Table S1). The alpha diversity was greatest for the samples of the production water (V20002_1, V48002b_1) and the sample of domestic wastewater (T53001), as evidenced by the high values of the Chao1 and Shannon indices.

**Fig 1:**
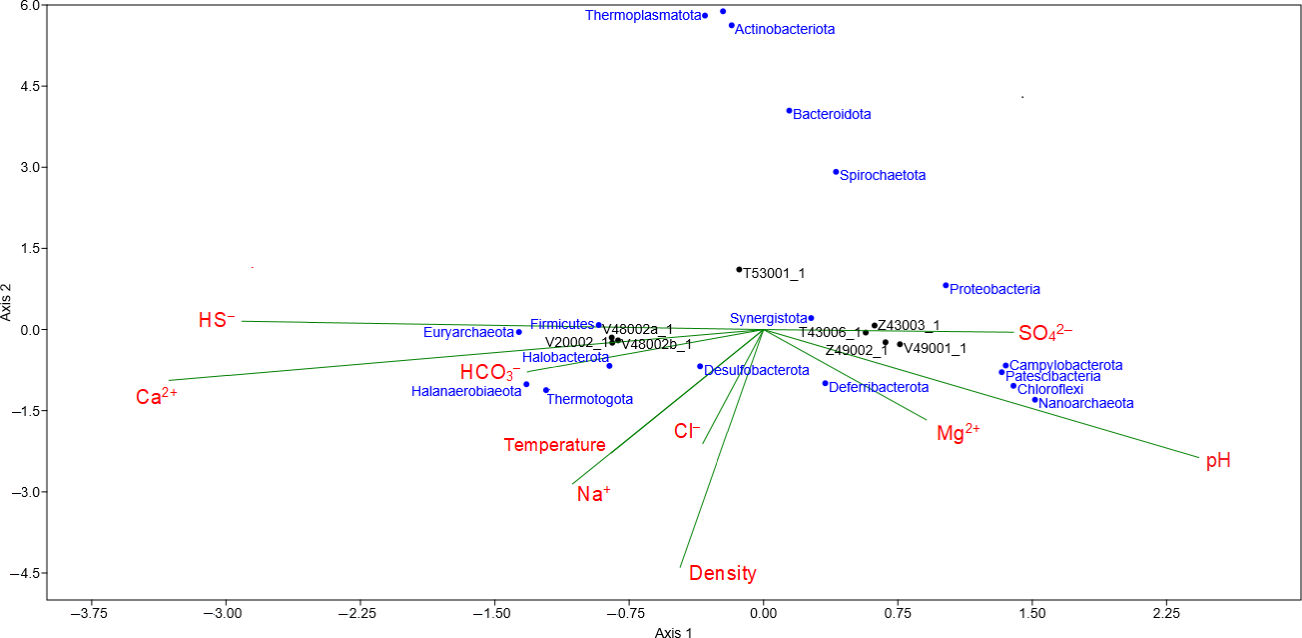
Canonical correspondence analysis (CCA) of the abundance of microorganisms at the phyla level and environmental parameters in the water samples collected in July 2023. The CCA graph is built using the program PAST 4.03 (52). The names of microbial phyla occurring in the water samples are indicated in blue. Physicochemical parameters of the samples are indicated in red. The directions of variation of environmental parameters in the samples are indicated by the green vectors.

The studied water samples were dominated by *Bacteria*. The proportion of *Archaea* did not exceed 8% (of the total number of sequences in the libraries) (Fig. S2). A comparison of the composition of microbial communities using the principal component analysis (PCA) method revealed the distribution of microorganisms into three groups (Fig. S3). The first group included microorganisms from the produced water collected at the outlet of the separator V20002 and at the inlet and outlet of the equalization tank V48002. Moreover, the microorganisms of water samples from the same sampling sites, taken in June 2023 and in January and November 2024, were grouped together, which indicates their relative stability. The second group consisted of communities of the wastewater tank of safe drains T53001, which received wastewater from safe zones, equipment, a helicopter pad, precipitation from roofs and open areas, as well as from a domestic wastewater treatment plant. The third group consisted of the remaining samples, which are a mixture of domestic wastewater, seawater, and production water. The distribution of microbial communities into three groups and the association of thermophilic bacteria of the phyla *Thermotogota* and *Desulfobacteriota,* and archaea *Euryarchaeota* with sulfide-containing water samples with high temperatures is confirmed by Canonical correspondence analysis (Fig. 1).

**Fig 2:**
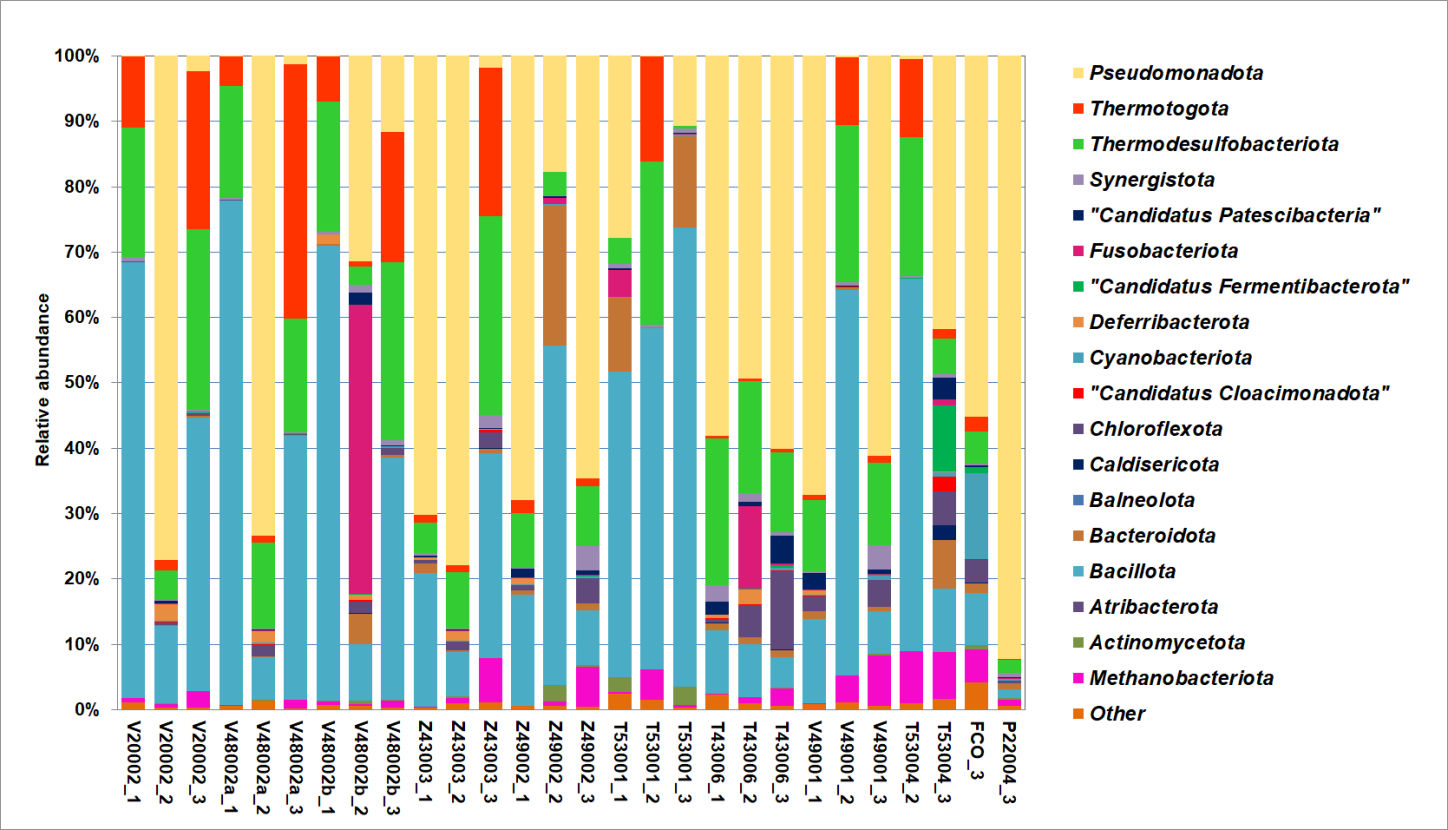
Relative abundances of prokaryotes at the phylum level revealed in water samples by V3–V4 fragments of 16S rRNA gene sequencing.

In the samples of reservoir water separated from oil from separator V20002, as well as in the water V48002a and V48002b, a microbial community typical of high-temperature oil formations with sulfate-containing water of increased mineralization was found. The samples taken in July 2023 and November 2024 were dominated by bacteria from the phyla *Bacillota* (37%–77% of the total number of sequences in libraries), *Thermodesulfobacteriota* (17%–27%), and *Thermotogota* (4.5%–38%). The bacteria of the phyla *Pseudomonadota* (0.1%–11.5%), *Synergistota* (0.1%–1.1%), and *Calditrichota* (0.1%–1%) were less represented in these samples (Fig. 2). However, in the samples taken in January 2024, the share of dominant taxa decreased in favor of mesophilic bacteria of the phylum *Pseudomonadota* (31.4%–77.1%) and emergence of bacteria of the phyla *Deferribacterota* (0.8%–2.5%) and *Chloroflexota* (0.5%–1.8%), which may be due to the cooling of the water treatment system due to extremely low winter temperatures reaching –56°C. The archaeal sequences in the produced water V20002_2 belonged mainly to methanogens of the phylum *Methanobacteriota* (genus *Methanothermococcus*, 7%). Minor components were archaea of the phylum *Nanobdellota* and *Thermoproteota*.

**Fig 3:**
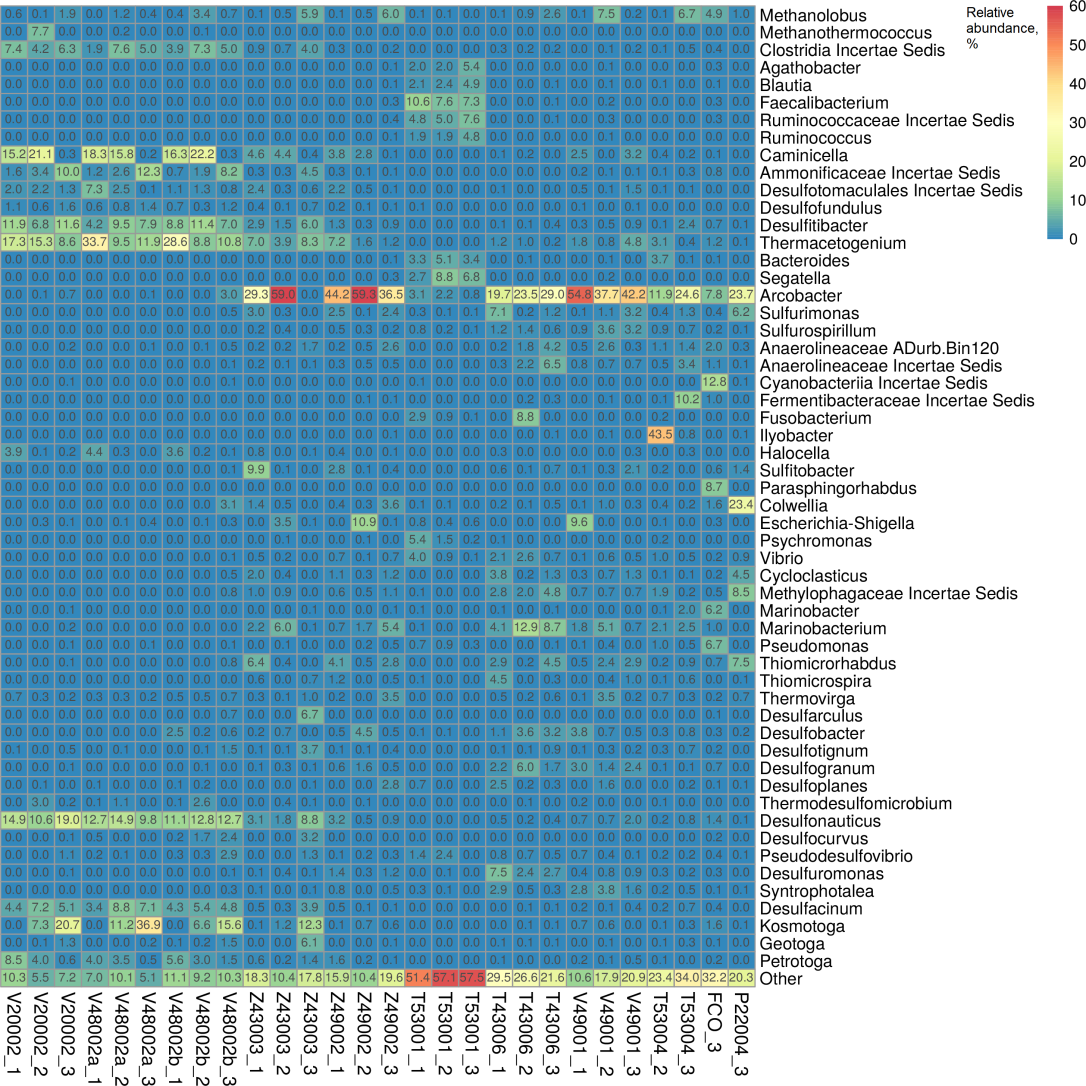
Heatmap based on 16S rRNA gene amplicon sequencing showing the relative abundance (%) of taxonomic groups of prokaryotes at the genus level in samples from the water treatment system in a dynamic environment.

Reservoir water from the equalization tank V48002 is mixed with the domestic wastewater from the T53001 safe wastewater tank, seawater, and ballast water coming from the oil tank, which is filled with seawater for fire protection purposes. These liquids flow sequentially into the tanks Z43003, Z49002, and Z49001 and are then pumped into the oil reservoir. The temperature of the liquid in front of the Z43003 gas cooler decreases to 22°C, which leads to the replacement of thermophilic SRB of the genera *Thermacetogenium*, *Desulfonauticus*, and *Desulfacinum* present in reservoir water with mesophilic SRB of *Desulfobacter* and *Desulfogranum* (Fig. 3).

Mesophilic bacteria of the oxidative branch of the sulfur cycle (14), including *Sulfurimonas*, *Sulfurospirillum*, *Sulfurovum*, and *Thiomicrospira*, were present in the water treatment system, and the representation of mesophilic bacteria of the genus *Desulfuromonas* capable of reducing elemental sulfur to sulfide in the medium with acetate increased in the T43006 water sample (63).

Fermentative bacteria of the produced water (V20002, V48002a, and V48002b) were dominated by thermophilic, anaerobic bacteria of the genus *Caminicella* of the phylum *Bacillota* (64), and bacteria with sheaths (toga) of the genera *Kosmotoga*, *Petrotoga*, and *Geotoga* of the phylum *Thermotogota* (65, 66). Thermophilic hydrogen-utilizing methanogens of the genus *Methanothermococcus* present in reservoir water were replaced by methanogens of the genus *Methanolobus* (Z43003_3, Z49002_3, and Z49001_2), preferring lower temperatures and methanol as a substrate (67, 68).

The microbial diversity in water samples taken after the FCO deep filter and after the P22004 pumps differed from that in other studied water treatment facilities by a greater representation of aerobic and facultative anaerobic bacteria present in seawater, such as *Colwellia*, *Arcobacter*, *Cyanobacteria*, *Parasphingorhabdus*, *Methylophagaceae*, *Pseudomonas*, *Marinobacter*, *Cycloclasticus*, *Thiomicrorhabdus*, as well as anaerobic methanogenic archaea of the genus *Methanolobus*. Dissolved oxygen present in injected seawater (1–3 mg/L; Table 1) can support bacterial growth on hydrocarbons coming from an oil storage tank, which is filled to the top with seawater to ensure fire safety.

Wastewater from T53001 was characterized by a wide variety of phylotypes and the presence of bacteria of the genera *Faecalibacterium*, *Subdoligranulum*, *Ruminococcus*, *Blautia*, *Bacteroides*, and *Vibrio*, indicating fecal contamination of these drains (69–71).

### Functional characterization of microorganisms in the water samples

Using the iVikodak program, the contribution of the studied microbial communities to the implementation of the main metabolic pathways was predicted according to the KEGG database (51). Figure S4 demonstrates the potential ability of microorganisms to carry out the main metabolic pathways—glycolysis/gluconeogenesis, the tricarboxylic acid cycle, and the pentose phosphate pathway, the metabolism of methane, nitrogen, sulfur, propanoate, and carbohydrates (starch, sucrose, fructose, and mannose). The greatest potential contribution to sulfur metabolism, which is important for the corrosion process, was made by reservoir water microorganisms, including the main groups of sulfidogens, *Thermacetogenium*, *Desulfitibacter*, *Desulfonauticus*, *Caminicella*, *Petrotoga,* and unidentified *Clostridia* (Table S2).

### RT-PCR of bacterial and archaeal 16S rRNA genes

The predominance of *Bacteria* over *Archaea* was shown using quantitative real-time PCR (qRT-PCR) of 16S rRNA genes in all studied water samples from the water treatment system at the Prirazlomnoye oil field (Table S3). In the produced water samples, V20002, V48002a, and V48002b, the number of copies of bacterial 16S rRNA genes ranged from 1.1·10^5^ to 4.9·10^8^ in 1 mL. The number of archaeal genes was relatively low—from 2.7·10^2^ to 3.8·10^5^ copies in 1 mL. The most polluted were samples T53001, T53004, and T43006, in which the number of copies of the 16S rRNA genes of bacteria and archaea reached 1.6·10^11^ and 4.1·10^7^ gene copies in 1 mL of water, respectively.

### Sulfide and methane formation by enrichment cultures from production water

The formation of sulfide and methane by microorganisms of V48002a_2 produced water, which was inoculated into media with different carbon and energy sources and electron acceptors, has been experimentally shown. The cultures were incubated at temperatures of 48°C and 55°C (Table 3). The sulfate present in the studied V48002a_2 produced water sample was reduced by almost all the obtained enrichments. Sulfide production was maximal in SRB cultures obtained on media with lactate/SO_4_^2-^ and acetate/SO_4_^2-^. The formation of methane was recorded in media with H_2_ + CO_2_, acetate, acetate/SO_4_^2-^, and acetate/H_2_ + CO_2_. However, the maximum accumulation of methane, reaching 7,300 ppm per vial, was observed in the medium containing iron (steel needle) as the only source of electrons, and CO_2_ as the carbon source. In the fermentative enrichment obtained in a medium with peptone and glucose, and in the culture of methanogens on a medium with acetate, the formation of small amounts of molecular hydrogen was recorded. In a minimal medium with Fe^0^, CO_2_, and sulfate, the formation of sulfide and methane by sulfate-reducing and methanogenic enrichments was registered, which indicates the use of iron as an electron source by SRB and methanogens simultaneously. However, the amount of methane formed by SRB enrichment was very low, which is consistent with the higher energy yield of the hydrogen consumption reaction by sulfate reducers, compared with methanogens, and confirms the known observation that sulfate reducers outcompete methanogens in sulfate-containing media.

**TABLE 3:** Methane and sulfide content in enrichment cultures of fermentative, sulfate-reducing, and methanogenic prokaryotes grown in media with different electron donors and acceptors over 30 days of incubation at 48°C and 55°C

Enrichment culture	Carbon source/electron donor	Acceptor	Gas phase	48°С		55°С	
				CH_4_, ppm	HS^–^, mg L^−1^	CH_4_, ppm	HS^–^, mg L^−1^
Fermentative	Peptone + glucose	SO_4_^2-^	Ar	0	475	0	389
	Pyruvate	S_2_O_3_^2-^	Ar	0	0	0	0
SRBs	Lactate	SO_4_^2-^	Ar	0	260	350	540
	Crude oil	SO_4_^2-^	Ar	0	155	Nd^*a*^	Nd
	Acetate/H_2_	SO_4_^2-^	H_2_	0	459	110	320
	CO_2_/Fe^0^	SO_4_^2-^	Ar/CO_2_	90	140	90	195
	Acetate	S^0^ + SO_4_^2-^	Ar	60	140	0	149
	Acetate	SO_4_^2-^	Ar	90	415	130	504
	CO_2_/H_2_	SO_4_^2-^	H_2_/CO_2_	0	314	85	201
Methanogens	CO_2_/H_2_	–^*b*^	H_2_/CO_2_	66	147	80	136
	Acetate	–	Ar	72	56	20	15
	Acetate, CO_2_/H_2_	–	H_2_/CO_2_	61	181	110	180
	CO_2_/Fe^0^	–	Ar/CO_2_	7,300	65	50	57
	Crude oil	–	Ar	90	149	Nd	Nd

^*a*^ Nd, no data.

^*b*^ “–” indicates that there is no acceptor in the medium.

**Fig 4:**
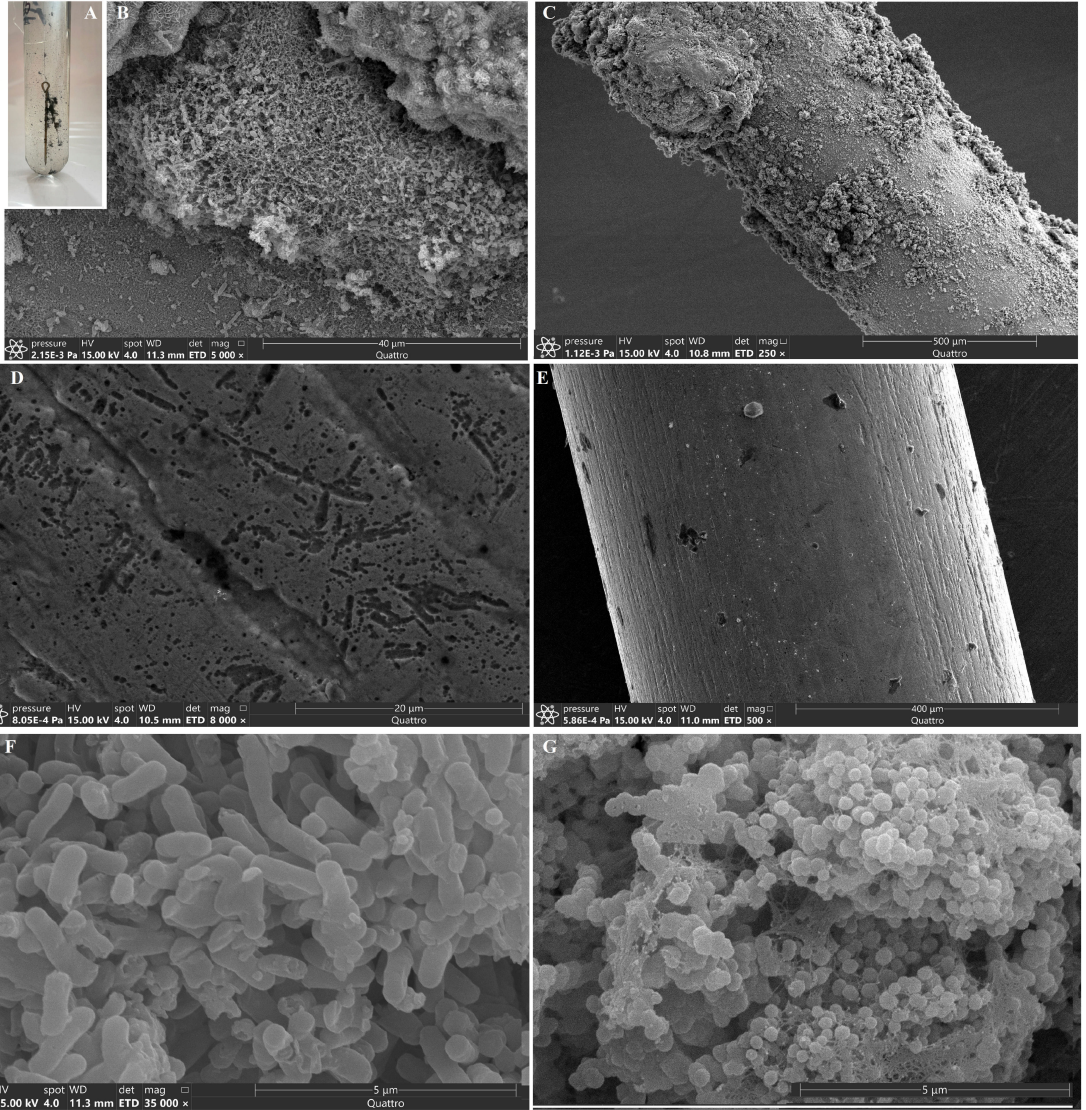
Biofilm formation in a medium for sulfate-reducing bacteria with a steel needle (**A**) and morphological diversity of cells in sulfate-reducing (**B**), fermentative (**F**), and methanogenic enrichments (**G**). The image of a steel needle with the pitting corrosion after removing biofilms and sulfide precipitation from it, and of a steel needle exposed in sterile medium for SRB is shown in panels **D and E**, respectively. The images in (**B–G**) were obtained using a scanning electron microscope (Quattro S, Thermo Fisher Scientific, Brno-Chernivtsi, Czech Republic) at an accelerating voltage of 15 kV.

All cultures formed abundant biofilms. In media with a steel needle, biofilms were associated with the needle (Fig. 4A). Figure 4B and C show the biofilms formed on a steel needle in the SRB enrichment. The needle was also coated with a black precipitate of iron sulfide, and pitting corrosion was shown after removing biofilms and sulfide precipitation (Fig. 4D). The steel needle exposed in the control sterile medium for SRB is shown in Fig. 4E. The morphological diversity of the cells was greatest in fermentative enrichments and included rod-shaped and coccoid cells of different sizes (Fig. 4F and G). Rod-shaped cells were connected by outgrowths resembling pili or electron-conducting filaments (Fig. 4B and G) similar to those previously described in SRB (35, 41, 72). The medium for autotrophic methanogens with H_2_/CO_2_ was dominated by coccoid cells morphologically resembling methanogenic archaea of the genus M*ethanothermoccoccus* (Fig. 4G).

## DISCUSSION

For a long time, the main standard method of controlling corrosive bacteria in the production waters of oil fields was the determination of the SRB numbers on a medium with lactate at 30°C. With the development of molecular ecological methods based on the analysis of 16S rRNA genes, functional genes, and metagenomes, the understanding of the diversity of corrosively active prokaryotes has significantly expanded. In this work, the diversity of microorganisms in water samples from different sites of the water and oil treatment system at the high-temperature Prirazlomnoye offshore oil field was studied.

To determine the sources of sulfide production and other possible corrosion agents of steel equipment, the key physicochemical parameters of production and injection water, the number of culturable sulfate-reducing bacteria, the number of bacteria and archaea (by qPCR), as well as the composition of microorganisms (by sequencing amplicons of the 16S rRNA gene fragments) were determined. Sulfidogenic bacteria, potentially capable of biocorrosion, were found in different sampling sites of the wastewater treatment system at the Prirazlomnoye offshore oil field, and the possibility of the involvement of SRB and methanogens in anaerobic corrosion of steel due to the use of Fe^0^ as an electron source was also shown.

There is extensive information on the microbial diversity of high-temperature oil reservoirs by analyzing 16S rRNA genes, as well as the isolation and description of thermophilic sulfidogens (13, 73–75). However, a relatively small number of articles are devoted to the study of corrosive prokaryotes from high-temperature oil reservoirs (7, 75, 76). The most active corrosion processes of steel equipment are observed in offshore oil fields developed from a platform where seawater with a high sulfate content is used for flooding (7, 76–78).

Sulfate-reducing bacteria and archaea are the most studied corrosion agents of steel equipment in oil fields. Using organic substrates or molecular hydrogen as electron donors, they reduce sulfate to sulfide, which reacts chemically with ferrous iron (Fe^2+^), producing FeS. The ability of hydrogenotrophic SRB, acetogens, and methanogens to perform electrochemical corrosion, taking up electrons directly from the metallic iron (Fe^0^) itself to reduce sulfate or CO_2_, respectively, significantly expands the range of possible corrosive prokaryotes and the conditions under which the corrosion process can occur (10–12, 23, 25–27).

Using the method of high-throughput sequencing of 16S rRNA gene fragments, thermophilic halotolerant microorganisms of the main metabolic groups associated with oil reservoirs were detected in the produced water, that is, sulfate-reducing, fermenting, syntrophic, and methanogenic prokaryotes. The production water contained sulfide (≈50 mg L^−1^), which was probably partially chemically oxidized in the water treatment system when it was mixed with seawater and wastewater containing traces of dissolved oxygen, as well as a result of the activity of bacteria of the genera *Sulfuriminas*, *Sulfurospirillum*, *Sulfurovum*, *Thiomicrorhabdus*, and *Thiomicrospira* (14, 79). The temperature of the produced water was 50°C–56°C and decreased to 22°C when mixed with seawater, which led to the emergence of metabolically diverse mesophilic bacteria in the water treatment system.

Thermophilic SRB of the genera *Thermacetogenium* (8.6%–33.7%), *Desulfonauticus* (9.8%–19.0%), and *Desulfacinum* (3.4%–8.8%) accounted for a significant part of the microbiota in the produced water samples V20002, V48002a, and V48002b. However, their share further decreased in the water treatment system, and in the injected V49001 water, it did not exceed 4.8%, 2.0%, and 0.4%, respectively (Fig. 3). On the contrary, the share of mesophilic SRB of the genera *Desulfobacter* and *Desulfogranum* increased.

Bacteria of the genus *Thermacetogenium* are obligate anaerobes, thermophiles capable of growing on acetate in the presence of methanogens (80). The type PB^T^ strain of the only described species of this genus, *Thermacetogenium phaeum*, is represented by gram-positive spore-forming rod-shaped cells, which grow optimally at a temperature of 58°C and a pH of 6.8. The bacterium grows in the absence of electron acceptors to form acetate from alcohols, methoxylated aromatic compounds, pyruvate, glycine, cysteine, and formate, and the ability to grow on H_2_/CO_2_ to form acetate allows these bacteria to occupy the niche of acetogenic bacteria in the food chain. *T. phaeum* strain PB^T^ also oxidizes acetate with sulfate or thiosulfate as electron acceptors. *Thermacetogenium* spp. were detected by molecular methods in water samples from a number of high-temperature oil reservoirs (30, 81–83).

Thermophilic autotrophic SRB of the genus *Desulfonauticus* were the second major group in produced water samples (V20002, V48002a, and V48002b). They were first isolated from the shells of mollusks sampled in the area of deep-sea hydrothermal vents in the eastern part of the Pacific Ocean at a depth of about 2,600 m (84). The bacteria are represented by rods that do not form spores and grow at a temperature range from 30 to 60 °C in the presence of 0%–5% NaCl. The type strain *Desulfonauticus submarinus* 6N^T^ uses only H_2_/CO_2_ and formate as electron donors and acetate as a carbon source. Sulfate, sulfite, thiosulfate, and elemental sulfur are used as electron acceptors during hydrogen oxidation. Bacteria *Desulfonauticus* spp. were also found in oil reservoirs in Germany (85) and in the Uzen oil field flooded by the water of the Caspian Sea (Republic of Kazakhstan) (35). Autotrophic bacteria of the genus *Desulfonauticus* probably do not grow on the Postgate medium with lactate and sulfate, which is recommended for monitoring SRB in oil fields.

The fermentative bacteria, detected in produced water samples, belonged to the genus *Caminicella*, which includes thermophilic, anaerobic, spore-forming, and heterotrophic bacteria. The only described species of this genus, *Caminicella sporogenes*, represented by one strain AM1114^T^ growing at a temperature of 45°C–65°C, pH 4.5–8.0, in the presence of 15–46 g of NaCl/L (64). This type strain is capable of fermenting a mixture of 20 amino acids, complex protein substrates, and carbohydrates with the production of H_2_, CO_2_, butyrate, ethanol, acetate, formate, and L-alanine. In the presence of elemental sulfur and thiosulfate, the strain formed sulfide. *Caminicella* spp. were detected by molecular methods in the oil reservoirs of Oman (86), as well as in the biofilms that cause corrosion of steel in the seawater supply system at an oil field on the North Sea shelf (87).

Bacteria of the genus *Desulfitibacter* (88), similar to *Caminicella*, are capable of fermenting a number of substrates, including lactate and methanol, reducing oxidized sulfur compounds and producing H_2_S and CO_2_, which causes their participation in the corrosion of steel equipment in oil fields along with SRB.

Fermentative bacteria in the produced water also consisted of bacteria with sheaths of the phylum *Thermotogota* (genera *Kosmotoga*, *Petrotoga*, and *Geotoga*), which are common inhabitants of high-temperature and low-temperature oil reservoirs (65, 66, 89). A number of representatives of this group are able to reduce oxidized sulfur compounds to form sulfide and participate in the corrosion process.

In the samples where the produced water was mixed with seawater (FCO, P22004, T43006, T53004, and V49001), the microbial community was enriched with facultatively anaerobic organotrophic bacteria of the genera *Colwellia* and *Arcobacter*, which had previously been isolated from seawater and marine sediments (90, 91). The type species of the genus *Colwellia*, *Colwellia hadaliensis*, isolated from the Puerto Rico basin in the Atlantic Ocean, grew at a maximum hydrostatic pressure of 1,020 atm at 2°C (90). The bacteria *Colwellia chukchiensis*, *Colwellia arctica*, and *Colwellia beringensis* isolated from seawater samples or marine sediments from the Chukchi and Bering Seas and the Arctic Ocean are psychrophilic or psychrotolerant, reducing nitrate to nitrite or to molecular nitrogen (92–94). Strains of *Colwellia marinimaniae* isolated from crustaceans extracted from the Challenger deepwater region in the Mariana Trench were obligately barophilic and grew at a hydrostatic pressure of 80–140 MPA at 6°C (95). Bacteria of the *Colwelliaceae* family were part of the hydrocarbon-oxidizing populations of seawater in the oil spill zone in the Gulf of Mexico (96).

Bacteria of the genera *Pseudomonas*, *Marinobacter*, *Cycloclasticus*, and *Parasphingorhabdus* are also found in seawater (97–102) and are capable of aerobic oxidation of petroleum hydrocarbons present in the studied water samples. The products of aerobic oil biodegradation can serve as substrates for anaerobic components of the microbial community, reducing sulfate from formation water and injected seawater, and thus contribute to sulfide formation and corrosion of steel equipment.

Bacteria of the *Deferribacteraceae* family and of the genera *Geotoga* and *Pseudoalteromonas* are able to reduce oxidized iron to Fe^2+^ and directly participate in the corrosion process (21).

Methylotrophic methanogens of the genus *Methanolobus*, identified in samples of the FCO, P22004, V49001_2, T53004_3, and Z49003_3 (1%–7.5%), were previously isolated from oil and gas fields, mangrove deposits, salt lakes, and estuarine sediments (https://lpsn.dsmz.de/genus/methanolobus, accessed on 1 April 2025). They could be supplied to the water treatment system from both seawater and ballast water from an oil storage tank. The source of methanol in the water treatment system could be biocides containing formaldehyde with methanol, which is confirmed by the greater presence of *Methanolobus* in water samples from biocide-treated tanks (V49001_2, T53004_3, and Z49003_3). *Methanolobus* spp. does not grow on hydrogen and is not capable of receiving electrons directly from metal. The degradation of methanol by methanogens of the genus *Methanolobus* may indirectly contribute to the corrosion process.

Methanogens of the genus *Methanothermococcus* were found in the produced water (V20002_2). The strain *Methanothermococcus* (=*Methanococcus*) *thermolithotrophicus* ST22, previously isolated from the oil field in the North Sea, grew on H_2_/CO_2_ in the temperature range from 17 to 62°C and salinity up to 9.5% (wt/vol) NaCl (67). Methanogens of the genus *Methanococcus* have been shown to have high corrosion activity upon direct contact with Fe^0^, which is due to increased glycosylation of extracellular (NiFe) hydrogenase, which promotes its fixation on cell surface proteins (103). In our study, the formation of methane by thermophilic hydrogenotrophic methanogens from reservoir water V48002a_2 in a medium containing iron (Fe^0^) as the only source of electrons and CO_2_ as a carbon source was demonstrated, which indicated their possible involvement in the corrosion of steel equipment at the oil field.

The microorganisms found in produced and injected water at the Prirazlomnoye field were functionally similar to those of thermophilic sulfate-reducing consortia enriched from offshore-produced water by Okpala et al. (104). These consortia also included sulfate- and sulfur-reducers (genera *Desulfoplanes*, *Deulfotomaculum*, *Desulfomicrobium*, and *Desulfuromonas*), methanogens (genera *Methanoculleus*, *Methanosaeta*, and *Methanosphaera*), acetogens (*Acetobacterium*), and fermentative bacteria (*Smithella*, *Halanaerobium*, and *Clostridia*/*Clostridium*). It is important that these consortia also carry out sulfate reduction in the absence of organic substrates and H_2_, using iron (Fe^0^) as an electron donor. Sulfidogenic and methanogenic microbial communities that carry out the corrosion process in high-temperature oil reservoirs need further research.

### Conclusions

The results of the study of the Prirazlomnoye oil field showed the presence of a unique corrosion-active microbial community potentially capable of both general and pitting corrosion of steel equipment. Sulfide entered the system with the formation water and was oxidized both chemically and biologically to oxidized sulfur compounds, which supplemented the pool of sulfates present in the produced and injected seawater. Thermophilic halotolerant SRB of the genera *Thermacetogenium*, *Desulfonauticus*, and *Desulfacinum*, as well as a population of fermenting bacteria of the genera *Caminicella*, *Desulfitibacter*, *Kosmotoga*, *Petrotoga*, and *Geotoga,* reducing sulfur compounds other than sulfate, were probably the main sulfidogens. They entered the system with reservoir water and were responsible for the general corrosion of steel equipment.

Hydrogenotrophic sulfidogens and methanogens could use iron (Fe^0^) and CO_2_ and carry out pitting corrosion under conditions of temperature and salinity typical for different sites of the water treatment system. The aerobic bacteria *Colwellia*, *Pseudomonas*, *Marinobacter*, *Cycloclasticus*, and *Parasphingorhabdus*, probably originating from seawater, oxidized the hydrocarbons of residual oil present in wastewater and supplied anaerobic microorganisms with easily recyclable substrates. Iron-reducing bacteria of the *Deferribacteraceae* family and the genera *Geotoga* and *Pseudoalteromonas* were probably also involved in pitting corrosion. Bacteria of the genus *Thermacetogenium* have wide metabolic capabilities when growing on H_2_/CO_2_ or other substrates. They could carry out sulfate reduction, perform the function of acetogens, producing acetate from H_2_ and CO_2_, as well as grow syntrophically with methanogens on acetate. They can be the main agents of general, pitting, and acid corrosion. Knowledge of the taxonomic composition of microorganisms of reservoir water and of the water treatment system will improve the methods of targeted monitoring of sulfidogenic bacteria. The isolated cultures of sulfidogens can be used to select effective biocides and corrosion inhibitors used in the oil field.

## Data Availability

The original sequencing data have been deposited at the National Center for Biotechnology Information (NCBI) Sequence Read Archive presented in the study are openly available in the NCBI Sequence Read Archive (SRA; available at http://www.ncbi.nlm.nih.gov/sra/) under the accession number PRJNA1247773.

## SUPPLEMENTAL MATERIAL

Supplemental material msystems.01161-25-s0001.pdf Figures S1 to S4; Tables S1 and S3.

Table S2 msystems.01161-25-s0002.xlsx Potential contribution to sulfur metabolism of microorganisms from water samples.

ASM does not own the copyrights to Supplemental Material that may be linked to, or accessed through, an article. The authors have granted ASM a non-exclusive, world-wide license to publish the Supplemental Material files. Please contact the corresponding author directly for reuse.
