# Supplementary material for: Molecular and cultural evidence of corrosive microorganisms in an offshore oil field in the Arctic (Russia)
Source: mSystems. 2025 Oct 24;10(11):e01161-25. doi: 10.1128/msystems.01161-25 (PMC12625739; doi:10.1128/msystems.01161-25)
Supplement: Supplemental material — Figures S1 to S4; Tables S1 and S3. [file msystems.01161-25-s0001.pdf]

Supplemental Material

Molecular and cultural evidence of corrosive microorganisms in an offshore oil field in the Arctic (Russia)

Diyana S. Sokolova [1], Anna A. Kruglova [2], Ekaterina M. Semenova [1], Tatiana A. Mayorova [2], Andrey V. Mardanov [3], and Tamara N. Nazina [1,*]

[1] Winogradsky Institute of Microbiology, Research Center of Biotechnology, Russian Academy of Sciences, Moscow, Russia
[2] Independent Researcher, Saint-Petersburg, Russia
[3] Institute of Bioengineering, Research Center of Biotechnology, Russian Academy of Sciences, Moscow, Russia
* Correspondence: Diyana S. Sokolova, sokolovadiyana@gmail.com, or Tamara N. Nazina, nazina@inmi.ru.

This file includes:

Figures S1 to S4

Tables S1 and S3

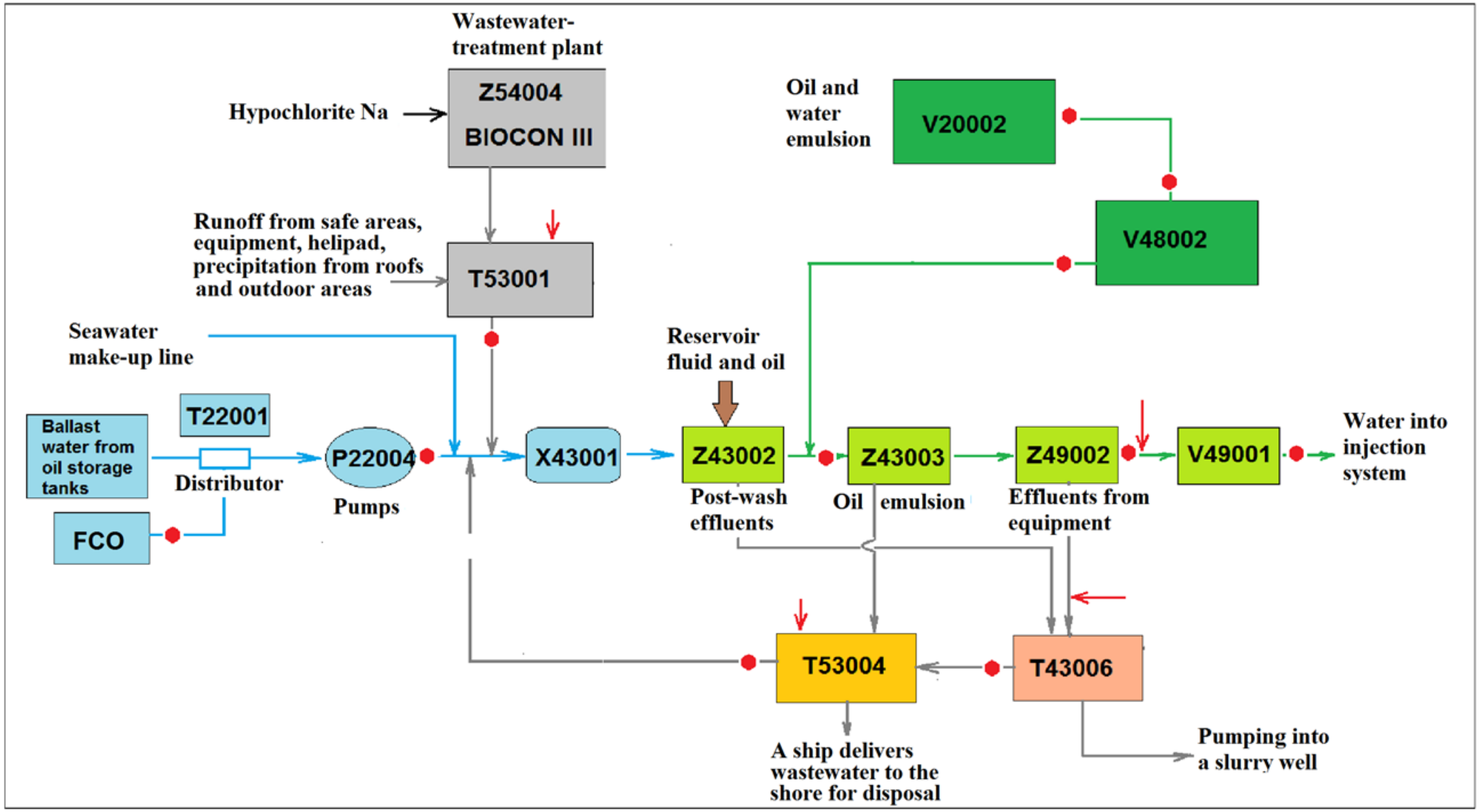

FIG S1 Schematic representation of the sampling sites (marked with red circles) from a water treatment system. Designations: V20002, separator of oil-water emulsion from the oil field; V48002, equalization tank with produced liquid; Z43003, gas flotation device; Z49002, fine filter; V49001, vacuum deaerator, preparing water for injection into the reservoir; Z54004, wastewater treatment plant; T53001, the tank with safe drains; distributor, directs the flows of ballast water from the oil storage tank and filtered water through the pump P22004 to the water treatment system;T53004, the tank with dangerous drains; T43006, the filter backwash tank; FCO, the coarse filter; P22004, pump. The red arrows mark the places where biocides enter the water treatment system. Tanks with production (formation) water are marked in dark green, seawater streams – in blue, domestic wastewater – in gray, and mixtures of seawater, household water, and production water are marked in light green.

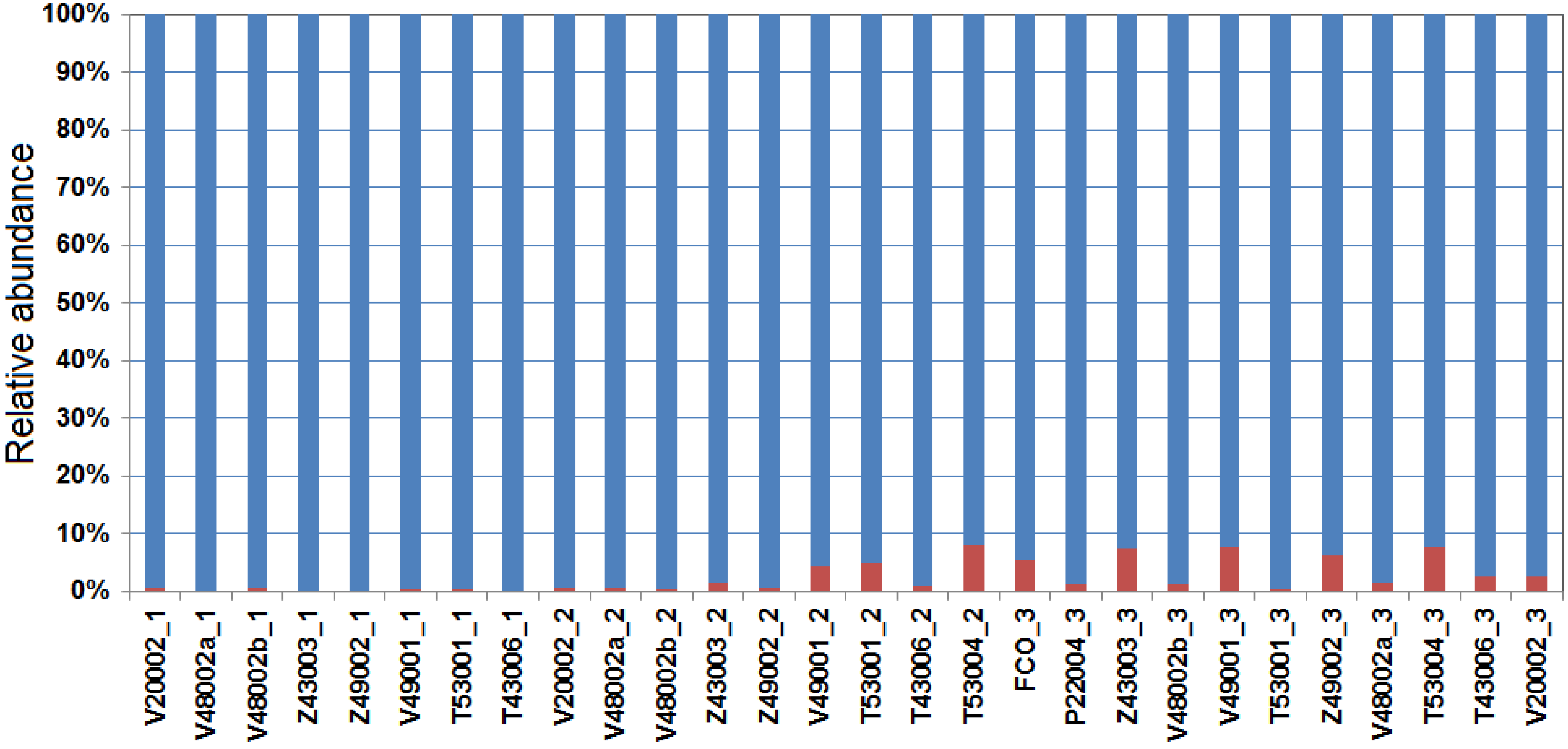

FIG S2 The relative content of V3–V4 fragments of 16S rRNA gene of *Bacteria* (blue) and *Archaea* (red) in libraries from water samples collected at the Prirazlomnoye oil field in June 2023 (designated _1), in January 2024 (designated _2), and in November 2024 (designated _3).

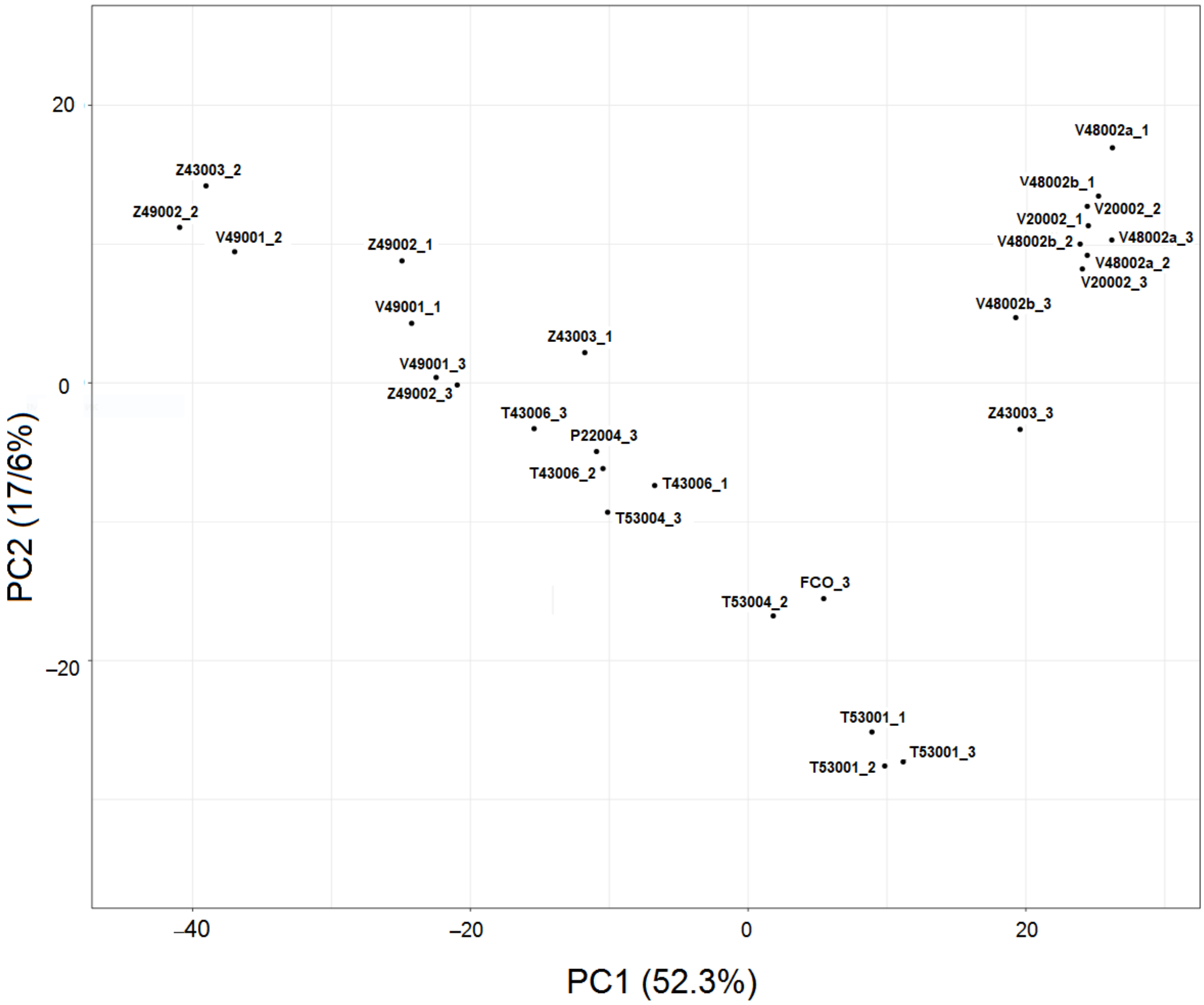

FIG S3 Comparison of the composition of microbial communities from water samples using the principal component analysis (PCA) method based on the relative abundance of operational taxonomic units including 16S rRNA genes (≥97% similarity) of microorganisms.

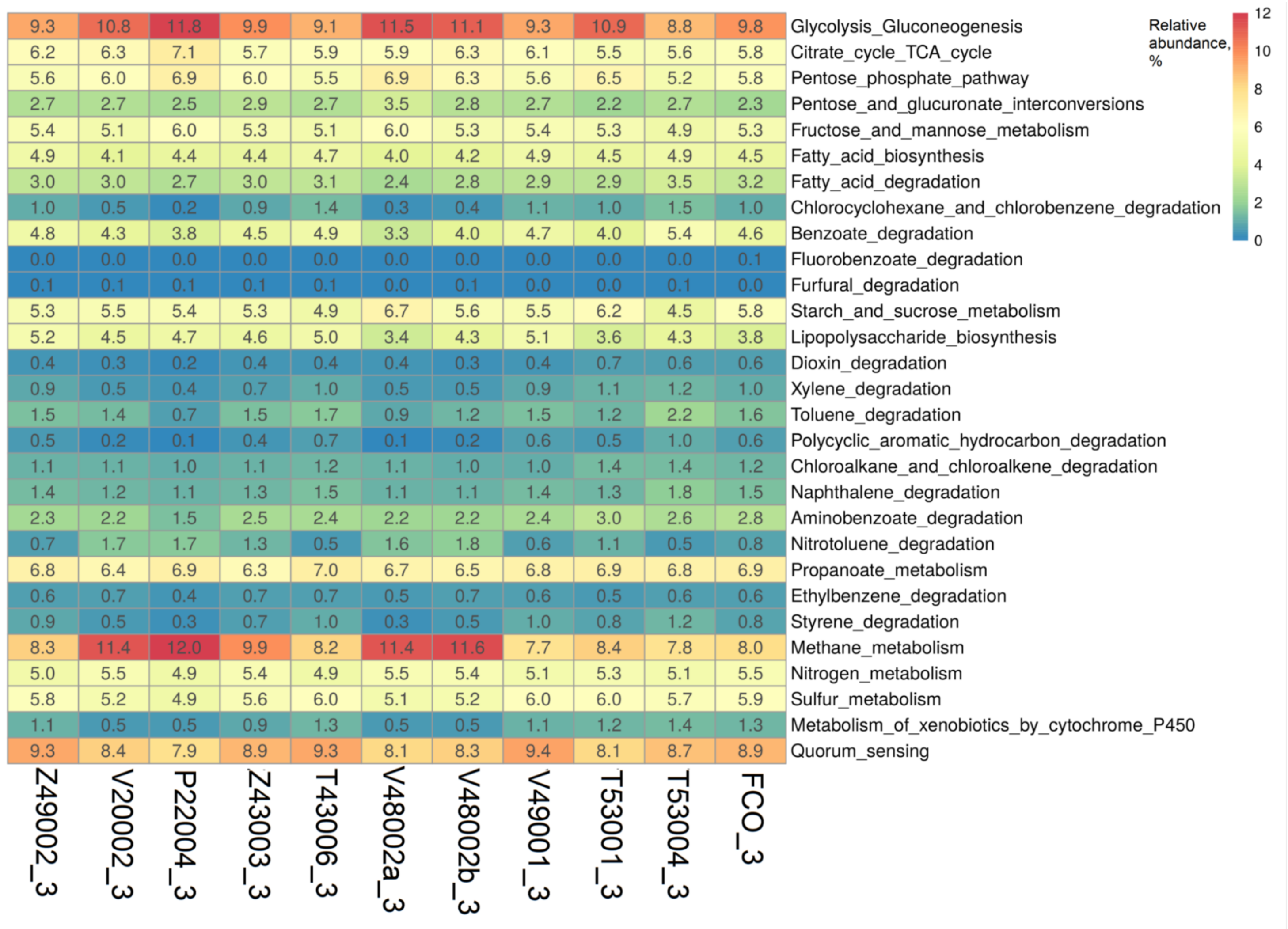

FIG S4 A heatmap showing the relative percentage of functional profiles of microbial communities from water samples taken at the Prirazlomnoye oil field, obtained using iVikodak.

TABLE S1 Alpha diversity indices based on the analysis of V3–V4 fragments of the 16S rRNA gene in libraries from water samples

Sample	Number of readings	Number of OTUs	bc-Chao1	Good's coverage	Shannon's H Index	Simpson's 1 D Index
V20002_1	31582	23459	269292.7	0.31	**9.51**	0.11
V20002_2	32590	4877	16931.11	0.89	5.55	0.11
V20002_3	13516	3130	16891.16	0.81	5.72	0.12
V48002a_1	20922	11900	92765.81	0.5	**8.49**	0.19
V48002a_2	35935	6703	25106.2	0.86	6.01	0.09
V48002a_3	11437	2204	13072.98	0.84	4.61	0.19
V48002b_1	30155	23235	308405.2	0.28	**9.62**	0.14
V48002b_2	31066	6232	24574.62	0.85	6.21	0.1
V48002b_3	14090	3443	16764.15	0.81	5.98	0.07
Z43003_1	38080	10539	57126.23	0.77	7.02	0.11
Z43003_2	31385	3309	14379.2	0.92	4.9	0.35
Z43003_3	15548	5170	29018.29	0.72	6.52	0.05
Z49002_1	50799	14340	74084.64	0.77	7.03	0.21
Z49002_2	21677	1999	7498.74	0.91	4.69	0.35
Z49002_3	26048	8539	46968.6	0.73	7.12	0.14
V49001_1	66277	30469	220456.2	0.6	8.65	0.19
V49001_2	20143	2228	8384.87	0.92	5.05	0.32
V49001_3	19509	6673	39285.22	0.71	6.74	0.16
T53001_1	49036	23681	167289	0.58	**8.83**	0.03
T53001_2	35965	16827	109790.9	0.59	**8.52**	0.03
T53001_3	21145	9470	59835.99	0.61	**7.94**	0.03
T53004_2	30332	8567	46773.14	0.77	6.35	0.2
T53004_3	19635	6596	45273.47	0.72	6.95	0.08
T43006_1	24004	14004	111036.4	0.48	8.61	0.06
T43006_2	33624	10854	50183.34	0.74	7.53	0.08
T43006_3	21177	6826	38176.46	0.73	6.85	0.11
FGO_3	26735	5372	26835.22	0.85	6.5	0.05
P22004_3	13741	3237	16328.84	0.81	5.7	0.13

TABLE S3 The number of 16S rRNA gene copies of *Bacteria* and *Archaea* in the studied water samples

No.	Sample	16S rRNA gene copy numbers of *Bacteria* per 1 mL of water			16S rRNA gene copy numbers of *Archaea* per 1 mL of water		
		**06.2023**	**01.2024**	**04.11.2024**	**06.2023**	**01.2024**	**04.11.2024**
1.	V20002	2.6E+07	4.1E+08	8.0E+06	9.0E+04	3.8E+05	1.2E+03
2.	V48002a	2.4E+07	4.1E+08	1.1E+05	2.9E+04	1.2E+05	2.7E+02
3.	V48002b	3.6E+07	4.9E+08	1.0E+07	8.8E+04	1.8E+05	2.5E+03
4.	Z43003	1.3E+06	9.3E+05	4.3E+07	1.0E+03	7.2E+02	2.9E+04
5.	Z49002	3.0E+07	3.3E+05	2.0E+10	8.2E+03	7.3E+02	7.0E+06
6.	V49001	1.8E+07	7.0E+05	4.1E+10	2.2E+04	5.7E+02	1.7E+07
7.	T53001	3.3E+07	2.8E+09	3.1E+10	-	6.8E+05	1.0E+06
8.	T53004	-**	3.3E+09	3.1E+10	-	1.6E+05	1.7E+07
9.	T43006	6.0E+07	8.5E+09	1.6E+11	-	7.2E+06	4.1E+07
10.	FGO	-	1.5E+03*	1.0E+06	-	-	2.7E+01
11.	P22004	-	9.4E+04*	7.9E+10	-	2.4E+03*	2.3E+06

*, The number of bacterial and archaeal sequences in 1 ng of DNA without conversion to sample volume. **-, No data.
